# Mr. Davies, on Obstetrical Cases

**Published:** 1810-01

**Authors:** H. Davies

**Affiliations:** Piccadilly


### *To the Editors of the Medical and Physical Journal.*

Gentlemen,

AMONGST the vast variety of obstetrical cases that fall to the lot of some Practitioners, it is a happy circumstance, that but comparatively few are of the *dangerous* kind; and, whenever these occur, it scarcely need be observed, that every effort with which an accurate knowledge of the science furnishes the Practitioner, should be early and invariably adopted, in order to rescue the patient from the impending danger.

Of all the dangerous labors, none, perhaps, requires a more prompt and decisive treatment, than those which are preceded by alarming hæmorrhages; occasioned either by a partial separation of the placenta from the uterus, or by its attachment over the os uteri.

In other cases of difficulty (if not danger), such as arise from unfavorable presentations of the fœtus, or mal-conformation of the pelvis, &c. the Practitioner has leisure to deliberate on the best mode of relieving his patient, without risking any consequences; but, in the *former*, it is generally found that "delays are dangerous;" and the sooner, without perturbation or confusion, an attempt is made to deliver, the greater probability there will be of saving our patient. It

It has been thought by some, after knowing the nature of the presentation, that if the os uteri was not at all, or but little dilated, the hæmorrhage not considerable, and the pulse good, that by waiting a longer period, the dilatation would gradually increase; and the hand, consequently, be introduced with more facility into the uterus. This has been known to be attended with serious consequences; for it ought to be recollected, that *should* the os uteri be disposed to dilate, with each dilatation a certain quantity of the vital fluid is lost, and thereby the patient so much the more endangered.

It will be readily granted, that an early attempt to dilate, in *some* cases, will be attended with manifest discouragement. The unyielding disposition of the os uteri, after the most patient perseverance on the part of the Practitioner, promises but little success. But what is to be done? The case is urgent, and without delivery, fatal. If but the smallest ground is gained in the attempt by gradually persevering, the delivery, in due time, will be accomplished; and, if undertaken in due time, the patient, for the most part, rescued from danger.

In some few cases it has been said, that the os uteri has been incapable of *any* dilatation whatever; and, that after a proper attempt having been made, it were useless to proceed, as in such a state it *never would* yield.

A deplorable case this, especially when it happens to be of the forementioned description! But, as nothing of this kind ever occurred in *my* practice (and I hope never will), I mention it as the sentiment of a celebrated Teacher in Midwifery, that others may judge of it as they think proper*.

If these cautionary hints may prove useful to the *junior* Practitioners in Midwifery, and tend, in any measure, to the welfare of such patients as are committed to their care, the end of their insertion in your useful Journal will be fully answered.

I am, &c.

H. DAVIES.

*Piccadilly*, 1809.

---

* The Editors know, from the best authority, that cases have occurred of placenta presentations, in which the *os uteri* did not become dilatable in time to save the life of the mother; and they think it the duty of every teacher of Midwifery, to apprise his pupils of the fact.

Obser-

